# Boosting piezoelectric properties of PVDF nanofibers via embedded graphene oxide nanosheets

**DOI:** 10.1038/s41598-024-66258-9

**Published:** 2024-07-17

**Authors:** Mahmoud Salama, Aya Hamed, Sara Noman, Germein Magdy, Nader Shehata, Ishac Kandas

**Affiliations:** 1 Center of Smart Materials, Nanotechnology, and Photonics (CSMNP), Smart CI Research Center, Alexandria University, Alexandria, 21544 Egypt; 2 Department of Engineering Mathematics and Physics, Faculty of Engineering, Alexandria University, Alexandria, 21544 Egypt; 3 Physics Department, Faculty of Science, Mansoura University, Mansoura, 35516 Egypt; 4 Department of Materials Science, Institute of Graduate Studies, and Research (IGSR), Alexandria University, Alexandria, Egypt; 5 Kuwait College of Science and Technology (KCST), 13133 Doha District, Kuwait; 6 USTAR Bio-Innovations Center, Faculty of Science, Utah State University, Logan, UT 84341 USA; 7 School of Engineering, Ulster University, Belfast, Northern Ireland BT15 1ED UK

**Keywords:** PVDF, GO, Electrospinning, PENG, Nanofibers, And SCPSs, Applied physics, Engineering

## Abstract

Tremendous research efforts have been directed toward developing polymer-based piezoelectric nanogenerators (PENG) in a promising step to investigate self-charging powered systems (SCPSs) and consequently, support the need for flexible, intelligent, and ultra-compact wearable electronic devices. In our work, electrospun polyvinylidene fluoride (PVDF) nanofiber mats were investigated while graphene oxide (GO) was added with different concentrations (from 0 to 3 wt.%). Sonication treatment was introduced for 5 min to GO nanosheets before combined PVDF solution. A comprehensive study was conducted to examine the GO incremental effect. Microstructural and mechanical properties were examined using a scanning electron microscope (SEM) and a texture analyzer. Moreover, piezoelectric properties were assessed via various tests including impulse response, frequency effect, d_33_ coefficient, charging and discharging analysis, and sawyer tower circuit. Experimental results indicate that incorporation of GO nanosheets enhances piezoelectric properties for all concentrations, which was linked to the increase in β phase inside the nanofibers, which has a significant potential of enhancing nanogenerator performance. PVDF-GO 1.5 wt.% shows a notably higher enhancing effect where the electroactive β-phase and γ-phase are recorded to be boosted to ~ 68.13%, as well as piezoelectric coefficient (d_33_ ~ 55.57 pC/N). Furthermore, increasing impact force encouraged the output voltage. Also noted that the delivered open circuit voltage is ~ 3671 V/g and the power density is ~ 150 µw/cm^2^. It was observed that GO of concentration 1.5 wt.% recorded a conversion efficiency of ~ 74.73%. All results are in line, showing better performance for PVDF-GO 1.5 wt.% for almost all concentrations.

## Introduction

There has been extensive research over the last decade on self-powered, intelligent, elastic, miniaturized, and sustainable new generations of electronic devices. A new era of revolution and significant efforts are exerted in developing self-charging powered systems (SCPSs) due to their effective role in smart wearable electronics^1,2^. SCPSs mainly depend on efficient energy-harvesting nanogenerators that convert the energies of the surrounding environment into electrical energy. SCPSs are a new trend in response to the global energy crisis and environmental pollution brought on by the increasing use of non-renewable energy sources. They are used in low-power electronic applications as they depend on various strategies for energy harvesting, including electromagnetic, electrostatic, piezoelectric, triboelectric, thermoelectric, and pyroelectric transduction mechanisms.

Piezoelectric nanogenerators technology was first proposed in 2006 and is based on converting ambient mechanical stress into electric power^3^. They are categorized into lead-based and lead-free piezoelectric materials. Pb_x_ Zr_1_-xTiO_3_ is known as Lead zirconate titanate (PZT) has a piezoelectric coefficient (d_33_ > 2500 pC/N)^4^.Furthermore, lead magnesium niobate/lead titanate (PMN-PT) composition has one of the highest known piezoelectric coefficients (d_33_ = 3140 pC/N)^5^. However, such materials still suffer from limited application due to its hazardous impact of Pb on both human health and the environment. So, in the near future, applications of pb-based materials will be severely constrained. According to that it is urgent case to gradually replace Pb containing materials and modify lead-free materials with high piezoelectric performance such as Barium titanate (BaTiO_3_) and zinc oxide (ZnO). Moreover, ferroelectric fluoropolymers especially polyvinylidene fluoride (PVDF) that proven to have appreciable piezoelectric coefficient values combined with good mechanical strength, thermal stability, and has excellent chemical resistance properties against different materials such as acids, bases, and organic solvents^6–10^.

PVDF attracted the attention of researchers a decade ago due to high energy conversion efficiency which can reach ~ 21.8% as reported by Chang^11,12^. In addition, the d_33_ coefficient which is in the range of 32 ± 1.73 pC/N^10^ is stated. Furthermore, PVDF is one of the commercially available piezoelectric polymers with light weight, durability, and flexibility. That made it a good choice for flexible piezoelectric nanogenerator to withstand human locomotion containing massive stress deformations like stretching, bending, and running^13^. Moreover, the fundamental structure of PVDF polymer chains is represented by a monomer unit comprising two carbon atoms, two hydrogen atoms, and two fluorine atoms, arranged as (CH_2_-CF_2_). It also has at least four crystal forms^14^. Three structures have permanent dipoles (β, γ, and δ phases) which depend on the sample preparation method. It is noteworthy that β-phase is the most polar one among all phases as all dipoles oriented in trans (TTTT) planner^15^, which makes it a desirable phase for piezoelectric energy harvesting.

Despite the importance of the electroactive β-phase and γ-phase, Piezoelectric performance of PVDF is enhanced via several factors. These factors include filler fraction, strain transfer to nanofillers, strain distribution around interface, and modulation of semicrystalline structures. Where other methods aim to enhance local dipole moment instead of enhancing the alignment (β-phase)^16,17^. Despite the advantages, the piezoelectric performance of PVDF is inferior compared to piezoceramics^18,19^. Hence, we aim to enhance the electroactive β-phase and γ-phase fraction by sophisticated design with addition of functional nanosheets and develop new smart fabrication methods.

Among all nanofiber fabrication methods, electrospinning is regarded as one of the lowest cost, simple structures, and most efficient syntheses for fiber. Electrospun nanofibers have excellent properties like light weight, porosity, surface area, and high aspect ratio, in addition to its scalability on an industrial scale^20^. Moreover, this method has been proven to have enhanced effect on piezoelectric properties of piezo nanofibers mats due to its process procedure depending on relatively high electric field which increases both dipoles alignment and β-phase fraction^21^. That leads to generate a remarkable voltage under mechanical stress, stretching, and human locomotion^12,22^.

Incorporation of nanofillers such as graphene oxide (GO)^23^, zinc oxide (ZnO)[24, 25]and ceramic based like barium titanate (BaTiO_3_)^26,27^ were proven to have a good impact on the piezoelectric properties of PVDF nanofibers. However, there is still a lack of research in GO proper treatment for optimum nucleation impact. In other literature, optimum concentration of added GO nanosheets was up to 1.6 wt.%^28^. Therefore, this paper aims at enhancing PVDF piezoelectric properties through incorporating GO nanosheets into the polymer matrix through introducing GO sonication treatment to ensure a significant reduction of GO size. Hence, PVDF-GO nanofibers mats fabricated with concentrations up to 3 wt.% leading to break of the maximum loading fabrication limitation found in other research work^29^.

**Figure 1:**
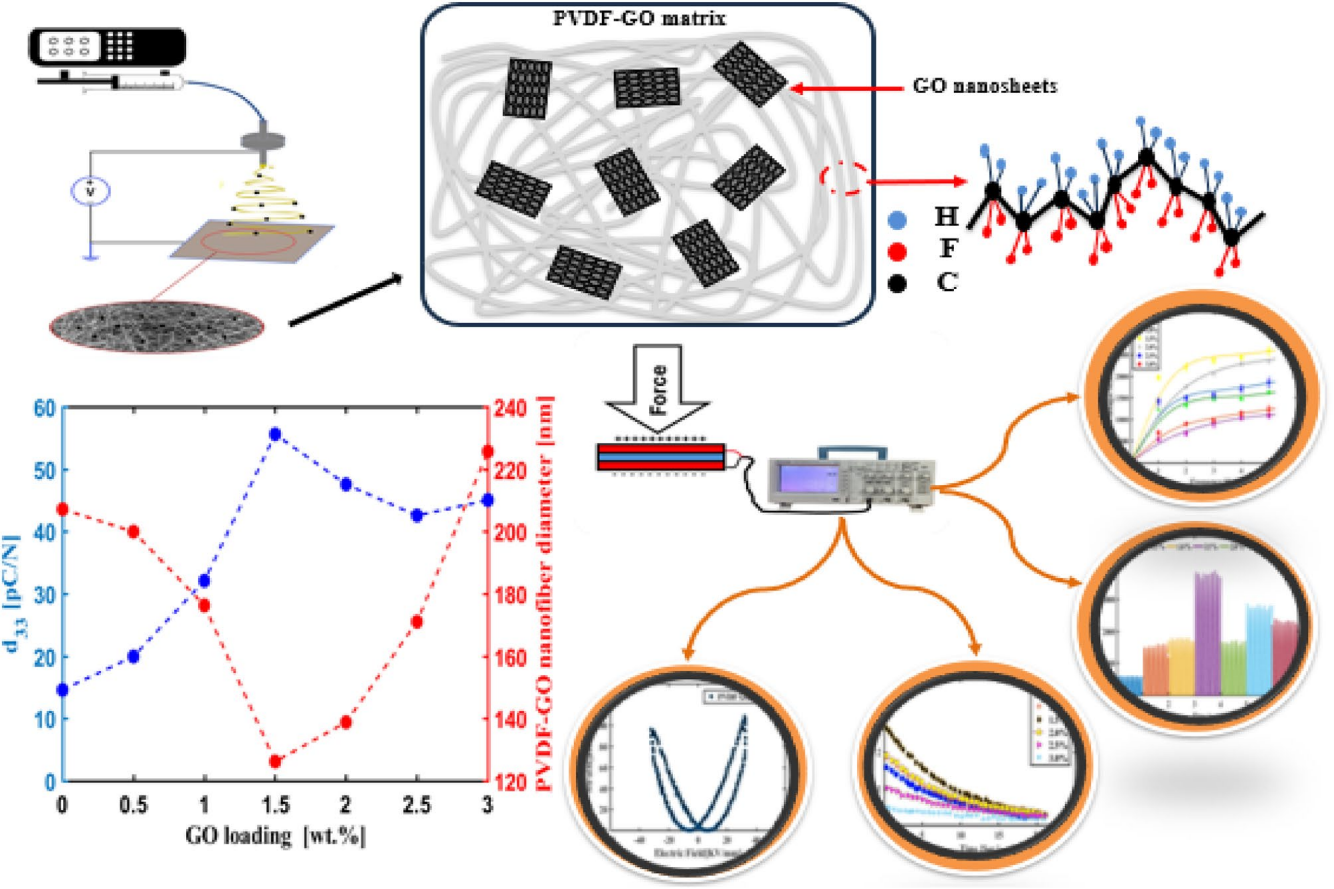
Nanofiber fabrication and characterization mechanisms for testing the piezoelectric performance of PVDF-GO wt.% samples.

GO acts as a nucleation agent that induces the electroactive β-phase and γ-phase formation and forms conductive path that enhance piezoelectric energy harvesting^22,30,31^. The optimum concentration of GO corresponding to highest piezo performance was investigated. Different GO concentrations in PVDF polymer were added to produce PVDF-GO electrospun nanofibers. The morphology and structure characterizations of the produced nanofibers were examined. The mechanical stability of the nanofibers mats was tested, and stress–strain curves were obtained for all produced mats. The piezoelectric performance of the nanofibers was thoroughly examined through different tests, for example impact test, d_33_, capacitance charging, and Sawyer tower circuit. Figure 1 shows an investigation of the effect of introducing GO into the PVDF nanofibers is done through different sets of tests to reach an understanding on how GO improves piezo performance of PVDF nanofibers such as developing the electroactive β-phase and γ-phase, improving d_33_ coefficient, enhancing mechanical properties, increasing open circuit voltage, and boosting electromechanical conversion efficiency.

## Materials and methods

### Materials

PVDF purchased from (Kynar®, King of Prussia, PA, USA). DMF solvent is obtained from (DMF 98%, Sigma Aldrich, Taufkirchen, Germany). GO nanosheets were synthesized from graphite with 5–20 μm grain size and was purchased from Fisher Scientific, UK, using the modified Hummer’s method^32,33^.

### Solution preparation

Dispersions of GO are prepared at different concentrations, ranging from 0.5 to 3 wt.%, by dispersing GO nanosheets within DMF solvent. Sonicating the mixture by probe sonication (Fischer Scientific-FB505) for 5 min ensures uniform dispersion of GO nanosheets. Besides, GO nanosheets were effectively obtained using probe sonication within the solvent matrix to facilitate their incorporation into the electrospun nanofibers^34^. This dispersion process is crucial for achieving homogeneous distribution of GO nanosheets throughout the polymer matrix, thereby influencing the final morphology and properties of the composite nanofibers^35^. Then, PVDF is dissolved in the solution with concentration 10 wt.%. The mixture is then stirred overnight at room temperature to create a well-blended and homogeneous solution.

### Electrospinning setup

**Figure 2:**
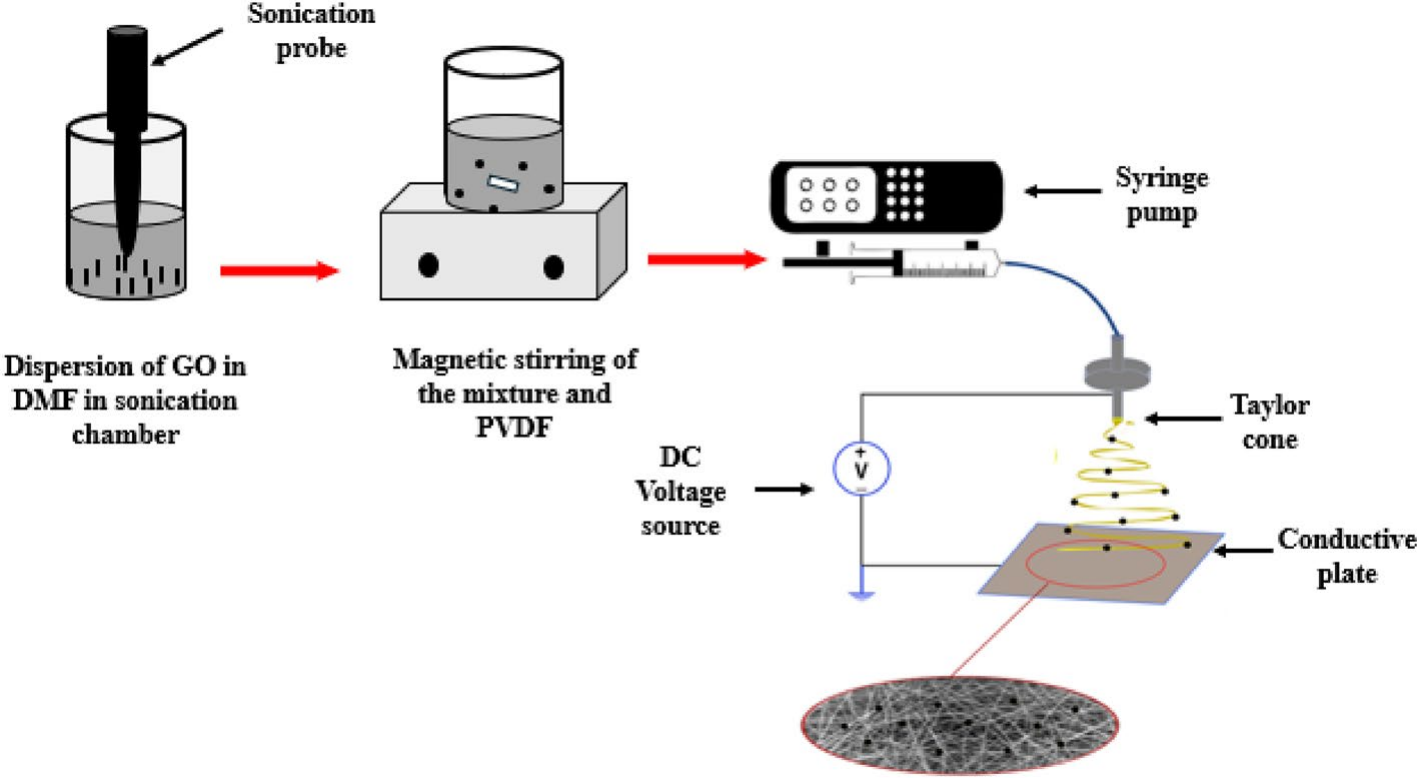
Schematic diagram of the different steps of composite fabrication, electrospinning setup and generated nanofibers.

Nanofibers synthesis is achieved through the electrospinning technique, due to its versatility and feasibility that enables the creation of nanofibrous structures with tailored properties. A syringe containing 6 ml of solution is attached to syringe pump that delivers the polymer solution at a precisely set feed rate of 1 ml/hr., ensuring a continuous and controlled flow. The electrospinning apparatus as shown in Fig. 2 features a high-voltage power supply connected to a spinneret, which applies a voltage of 22 kV. This voltage induces the formation of Taylor cone at the spinneret's tip, where the polymer solution emerges^36^. The electrostatic forces generated result in the stretching and thinning of the polymer solution, ultimately forming nanofibers. These nanofibers are collected on a grounded collector, typically a metal plate, positioned at a specific distance of 15 cm from the spinneret. The random distribution of fibers is a common result of traditional electrospinning methods. This randomness is influenced by several factors, including the electrostatic forces applied during the process, the type of collector used, the properties of the electrospinning solution, and environmental conditions. These factors contribute to the fibers laying down in a random manner on the collector surface^37^.

### Morphological and physical characterization

Various morphological tests had bSeen performed to ensure that the resulting PVDF-GO nanofibers exhibit sophisticated morphological and structural characteristics. The morphology of electrospun nanofibers was assessed using scanning electron microscopy (SEM) (JEOL, JSM-6010LV-SEM, Tokyo, Japan), where a slice of sample coated with a thin layer of gold for conductivity purposes allowing for better imaging and analysis, as well as improving the resolution and contrast of the SEM images, providing clearer details of the sample morphology^38^, then a quantitative analysis was done on the average fiber diameter and its deviation. Utilizing Image-J to measure the fiber diameter (Madison, WI, USA). Fourier transform infrared spectroscopy (FTIR) (FT-IR; Vertex 70 FT-IR, Bruker, Billerica, MA, USA) was used to confirm and quantitively calculate the electroactive β-phase and γ-phase content in the formed nanocomposite mats.

### Mechanical characterization

To assess the mechanical characteristics of our fabricated nanofibers mat and how its tensile strength is impacted by the process factors, a tensile test was carried out at room temperature on PVDF-GO nanofibers membranes using the Texture Analyzer CTX testing device (AMETEK Brookfield, Middleboro, USA) with a strain rate of 10 mm/min, a zero-starting load, and a 50 N load cell. The samples were sliced into 10 mm × 40 mm rectangle-shaped strips. Each sample was kept in a holding frame made of two layers of cardboard that was 20 mm × 20 mm on the inside and 30 mm × 40 mm on the outside. Three samples from the same environment were tested for the nanofibrous to determine the average value.

### Piezoelectric characterization

For evaluating piezoelectric properties of PVDF-GO at different concentrations of GO nanosheets, numerous tests are performed including impulse, frequency response, d_33_, capacitance charging, and sawyer tower circuit. These tests would be clarified in detail as follows:

#### Impact test

**Figure 3:**
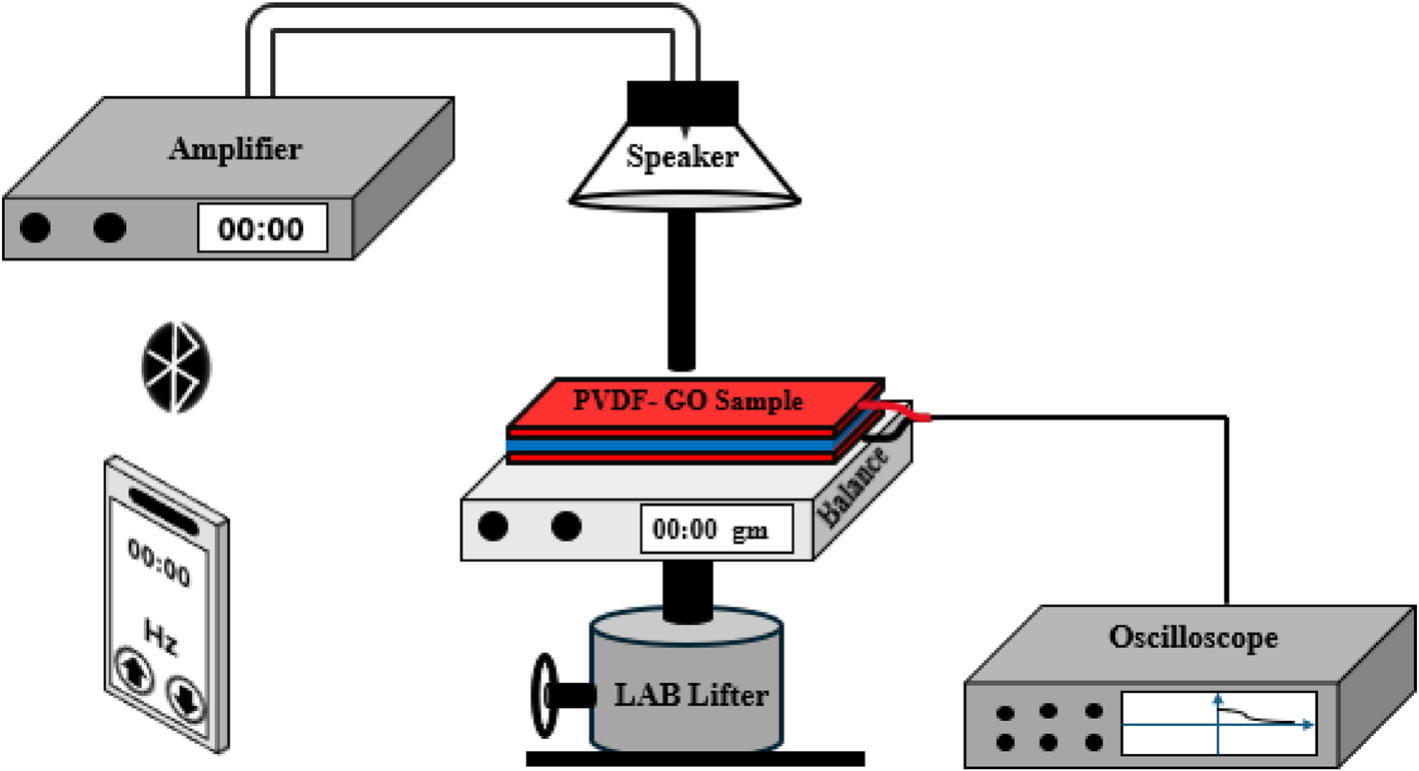
Configuration for cyclic piezoelectric tester that is used to generate controlled periodic force.

This test is mainly dependent on tunable force and frequency to measure the response and prove the enhancement of piezoelectric behavior of fabricated samples. A simple setup is designed that would help get accurate and systematic results with less effort. This setup includes loudspeaker, amplifier, plastic tip, balance, high impedance oscilloscope (Tektronix MDO3014), and LAB lifter as shown in Fig. 3. This new and simple setup provides variable frequency and force too. Hence the effect of their change can be studied accurately by doing force—voltage test at constant frequency and frequency-voltage test at constant force as well. To ensure fair comparison among samples, we have taken an average of nine measures. Firstly, we have maintained consistent sample area (20 mm × 40 mm) across all experiments to eliminate any potential bias arising from this factor. Additionally, we have utilized SEM measurements to characterize fiber diameter and mechanical properties, providing insights into their influence on the output voltage. Furthermore, we measure the GSM but while all the samples have the same area, we have normalized our data by dividing the output voltage by the mass of the sample only because it’s only the remaining parameter for us that will affect the results. This normalization approach effectively accounts for variations in sample mass, allowing for a more accurate comparison of results across different samples. The nanofibers sample is sandwiched between two conductive materials that are connected to oscilloscope to monitor the response where the speaker is driven by amplifier to have fair level of vibrations. The amplifier is controlled by mobile application to get monotone frequency in the form of square wave signal to drive the speaker. A tip is connected to the speaker to vibrate as a hummer with variable frequency. On the other hand, the force and consequently the pressure could be changed by regulating the amplifier output voltage as well as it can be achieved by regulating the distance between the sample and the tip through lifter, during this process the resultant average impact force value can be monitored through the balance screen.

Where the balance used to measure the mass, it’s noteworthy to mention that the average impact mechanical load is calculated according to Eq. (1):

1 $$F=M\times g$$

where M is the measured mass via balance and g is the gravity.

#### Piezoelectric coefficient (d_33_) test

**Figure 4:**
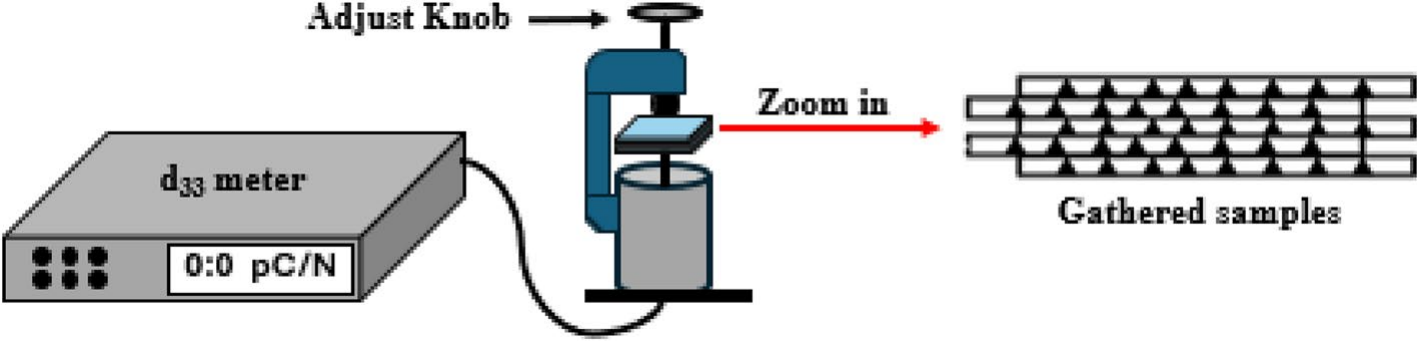
Configuration of d_33_ tester and samples arrangement in same direction.

To estimate the d_33_ coefficient of PVDF-GO at high resolution with high degree of reliability, samples are examined using Sinoceramics piezo d_33_ test system (YE2730, USA). Depending on the sample's thickness, we gather from 5 to 10 layers of the sample and stack them on top of one another while moving in a similar way to ensure the constructive dipole orientation as shown in Fig. 4, then measure the value many times. Following that, the average of 15 readings is then calculated.

#### Capacitance charging analysis.

**Figure 5:**
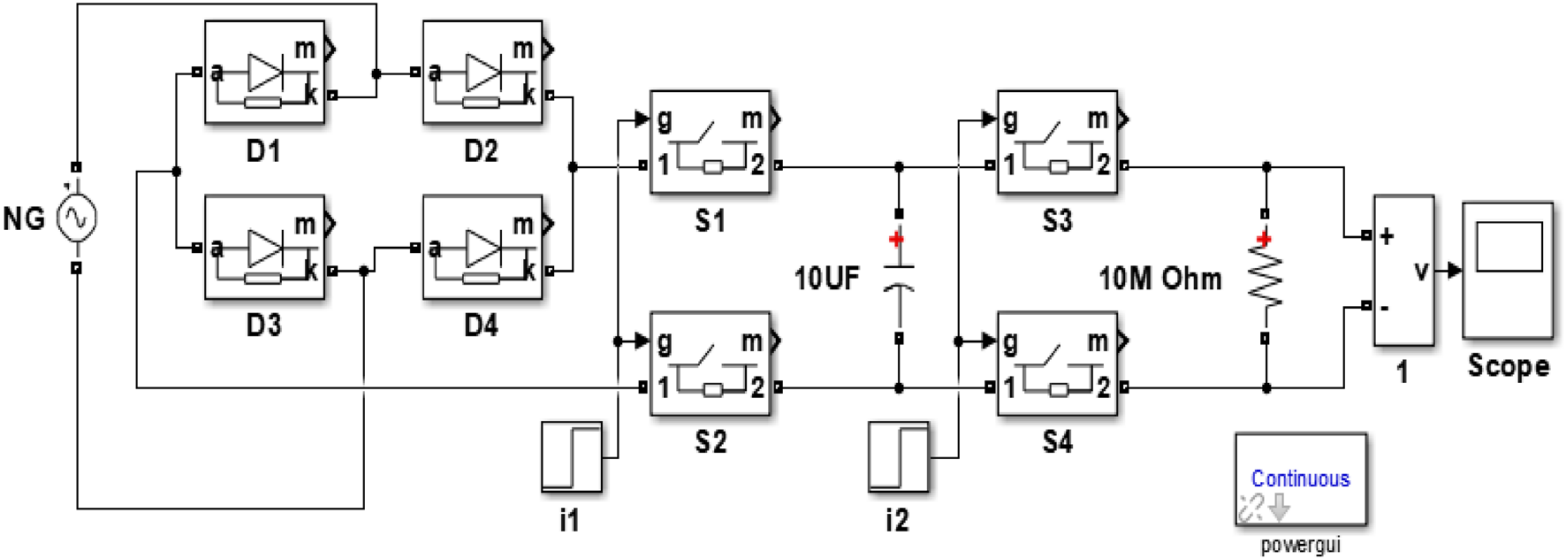
Schematic diagram of capacitance charging/discharging circuit with controlled simultaneous switches.

In this test we aim to check the ability of fabricated samples to act as a heart of nanogenerator producing large amount of energy and charge too. The sample is examined under continuous impact load at frequency 10 Hz and with two different loads 0.2 N and 0.4 N for period of 1 min. The output voltage is rectified through bridge that built using diodes and connected to 10 µF capacitor which accumulates the generated charges during the test. After that, we disconnect the source through simultaneous switches to ensure no more charge transfer. Then, the capacitor discharges its charge through a 10 MΩ resistor using another pathway through simultaneous switches as shown in Fig. 5. It is noteworthy to mention that the generated energy could be calculated from Eq. (2):

2 $$E=\frac{1}{2}C{V}^{2}$$

where C is the capacitance and V is the maximum voltage on the capacitor^39^.

#### Sawyer Tower circuit

**Figure 6:**
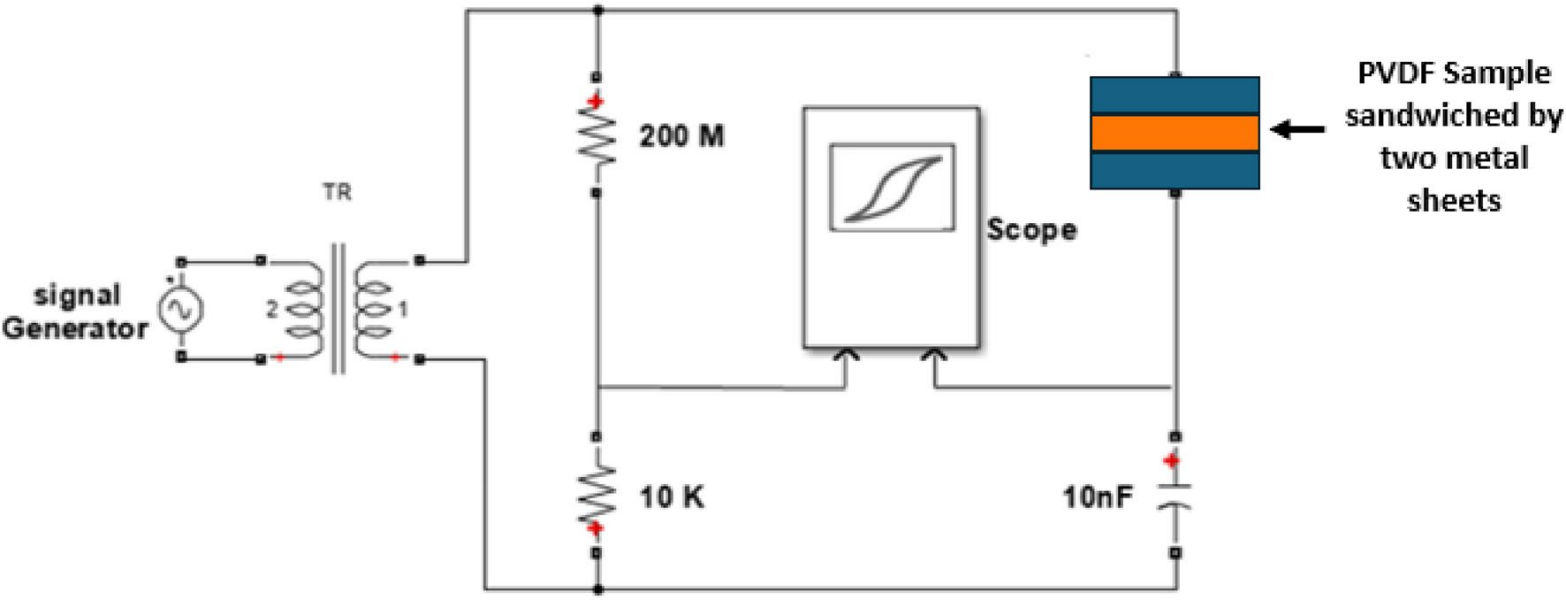
Schematic of Sawyer Tower circuit.

One of the most important characteristics of ferroelectric materials is polarization - electric field P–E hysteresis loop. It represents the real time polarization as a function of external electric field which helps in determining the ferroelectric properties such as dielectric constant and electromechanical coupling coefficient. Then, data could be extracted from it to generate strain-electric field S–E loops as they are clearly important in determining piezoelectric coefficient, d_33_ as well as determining the generating efficiency^40^. Hence, piezoelectric enhancement via addition of GO nanosheets with different concentration could be noticed. The Sawyer–tower circuit is shown in Fig. 6 is used to indicate the hysteresis loops of ferroelectric material where two capacitors are used as illustrated; the first one represents the sample capacitance, while the other one is a reference capacitor of much larger value^41^. The applied voltage is up to 2.5 kV at applied frequency of 5 kHz, where the voltage of signal generator has been amplified using step up transformer. The generated data then analyzed to calculate the applied electric field according to equation (3):

3 $$E=\frac{{V}_{in}}{d}$$

where ${V}_{in}$ is the applied voltage, and d is the sample thickness.

Now, the electric polarization P is calculated according to Eq. (4):

4 $$P=\frac{Q}{A}$$

where A is surface area, and Q is the charge on the sample capacitor.

Hence, we obtain P–E curve via high impedance oscilloscope (Tektronix MDO3014) then the data is analyzed aiming to obtain strain field loop S–E by squaring the polarization data. This loop has vital rules in designing generation and actuation applications and calculating the piezoelectric conversion efficiency. The efficiency can be calculated by considering the areas that drawn by S–E curve where it is a ratio between energy generated density represented by the area enclosed with strain axis and total applied energy considering losses that indicates by area inside the curve.

## Results and discussion

### Nanofibers characterization

**Figure 7:**
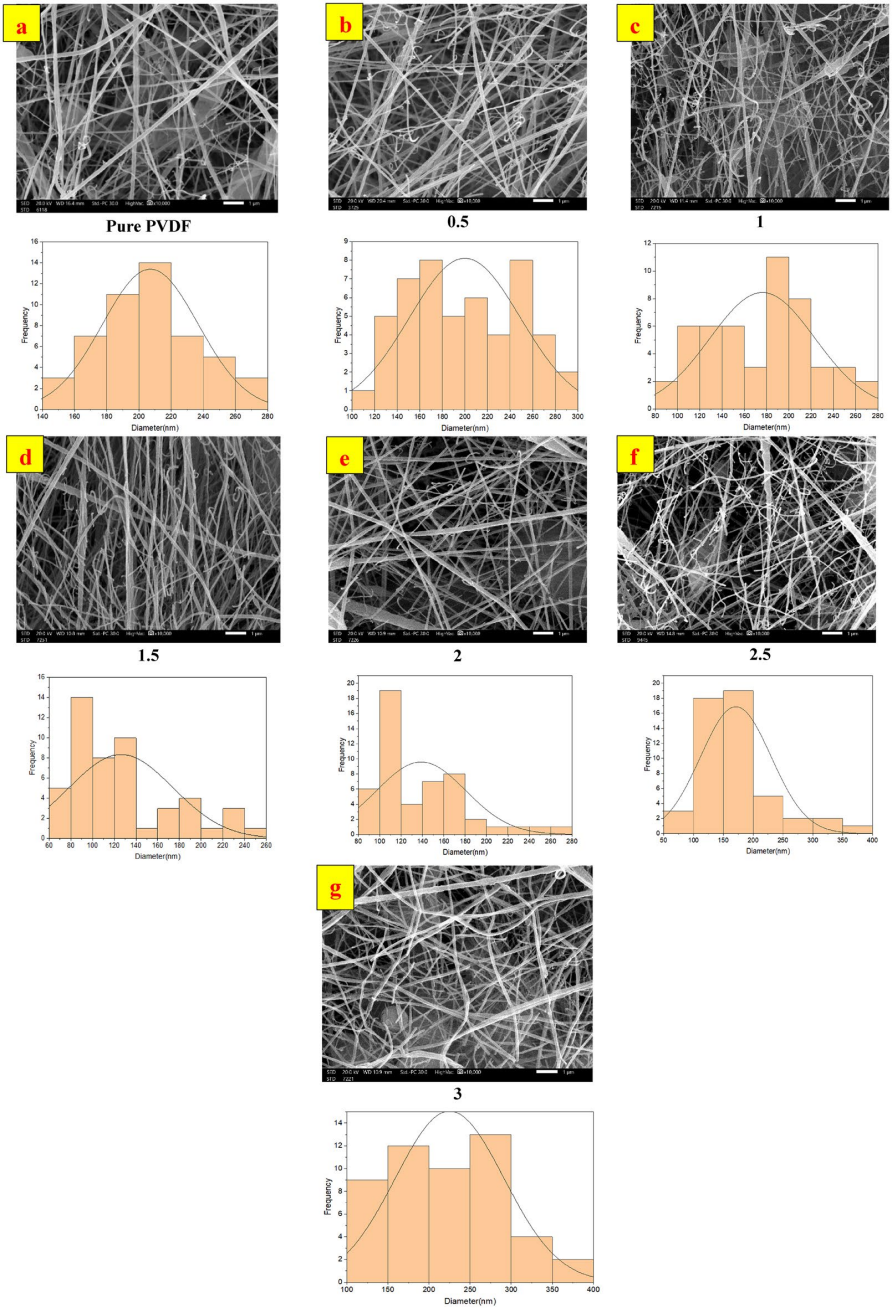
SEM micrographs of nanofibers, and composite nanofibers (**a**) Pure PVDF, (**b**) PVDF-GO 0.5wt.%, (**c**) PVDF-GO 1wt.%, (**d**) PVDF-GO 1.5wt.%, (**e**) PVDF-GO 2wt.%, (**f**) PVDF-GO 2.5wt.%, and (**g**) PVDF-GO 3wt.%.

The SEM images of PVDF-GO nanofibers at various GO concentrations (ranging from 0 to 3 wt.%) are displayed in Fig. 7, together with the related fiber diameter distribution. The fiber diameters are shown in SEM images and in the acceptable rang as it is varying between 100 and 500 nm in literatures^42,43^. SEM images revealed bead-free fibers and a normal distribution of fiber diameter, indicating that the addition of GO had no detrimental effect on fiber shape. Table 1 illustrates the rather clear influence of GO concentration on lowering nanofiber diameter. Fiber diameter generally decreased with more GO added, with an exceptional reduction can be seen at 1.5 wt.% GO. The enhanced electrolyte property and dielectric nature of GO are the primary causes of this decrease. Because of this, the addition of GO caused the blended solution to have more ions with a higher charge density. These ions would then be transported by the electrospinning jet and boost the solution's conductivity. In comparison to pure PVDF, this may result in an electrospun jet that is more stretched and PVDF-GO nanofibers with a lower produced mean diameter. However, with an increase in the GO percentage, the nanofiber diameter exhibits a subsequent rise. The reason for that, at lower concentrations of GO, the stretching and elongation of the electrospinning jet are enhanced, promoting the formation of thinner nanofibers^44^. This is likely due to the favorable interaction between the polymer chains and a moderate amount of GO, facilitating the electrospinning process. However, beyond a certain optimal concentration, as exemplified at 1.5 wt.% GO, the presence of an excessive amount of GO can disrupt the solution properties. This disruption may lead to changes in the rheological behavior of the solution, affecting the stability of the electrospinning jet and causing an increase in the diameter of the nanofibers^45^. Therefore, the concentration of GO should be carefully controlled to achieve the desired balance between enhanced stretching and the prevention of excessive disruption to obtain nanofibers with the desired diameter.

**Table 1:** PVDF/GO diameters of nanofibers samples with different GO concentrations.

GO concentration (wt.%)	Diameter (nm)
0	207.1 ± 29.48
0.5	199.93 ± 48.71
1.0	176.25 ± 46.78
1.5	126.17 ± 47.32
2.0	138.8 ± 41.20
2.5	171.03 ± 58.53
3.0	225.02 ± 65.60

**Figure 8:**
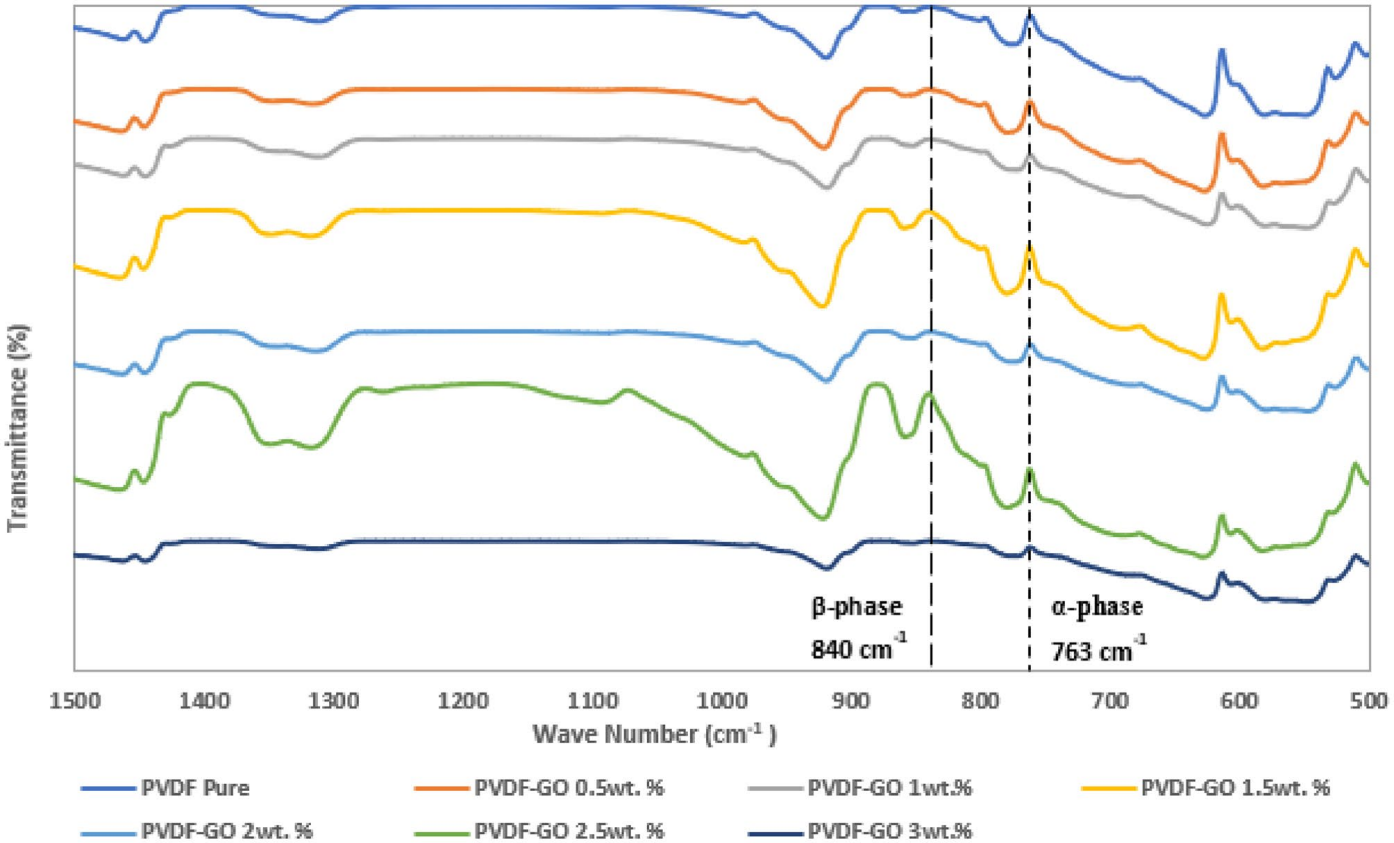
FTIR spectra of PVDF-GO samples with different GO concentrations.

The FTIR spectroscopy serves as a valuable tool, providing insights into different electroactive phases of PVDF. PVDF typically exhibits five crystalline phases (α, β, γ, δ, and ε), with the α-phase being the most prevalent non-polar phase, while the β-phase significantly contributes to enhancing piezoelectric properties. It was observed that electrospinning process has a positive impact on the piezoelectricity of the PVDF due to the high electric field effect on aligning the dipoles^46–48^. As shown in Fig. 8, The characteristic peaks for pure PVDF were observed at 1401 cm^−1^ which is associated with CH_2_. Two spectra bands at 1167 cm^−1^ and 1070 cm^−1^ were assigned for the asymmetric and symmetric stretching vibration of CF_2_, respectively. While CF_2_ bending vibration bands were recognized at 601 cm^−1^, the bending of C–H was observed at 875 cm^−1^. There are two asymmetric stretching vibration bands that were related to C–C–C and C–F at 837 cm^−1^ and 772 cm^−1^, respectively^49–51^. Nevertheless, the main characteristic bands for GO were observed at 1625, 1049, 951, and 821 cm^−1^ infers about the C=C, C–O (epoxy) and C–O (alkoxy) groups are confirmed from peaks respectively, and the peak at 1723 cm^−1^ corresponds to the carboxyl group^32^. So, it implies the successful formation of GO. However, these functional groups were not clearly observed from the neat PVDF nanofiber and PVDF-GO nanofiber composite membranes, probably due the small amount of GO content (e.g., ≤ 3% maximum) and the limited sensitivity of FTIR^52^.

FTIR spectroscopy is a very affirmative tool to collect information about different electroactive phases of PVDF. Figure 8 shows the FTIR spectra of PVDF, and PVDF-GO. For PVDF, a characteristic peak positioned at 618 cm^−1^ corresponds to the nonpolar α-phase, whereas the vibrational bands observed at 1275 cm^−1^, 840 cm^−1^, 510 cm^−1^, and 473 cm^−1^ affirm the presence of electroactive β-phase. In our calculations, peaks at 763 cm^-1^ are indicative of the α-phase while the 840 cm^-1^ FTIR peak is commonly associated with the electroactive β-phase and γ-phase in PVDF, as reported in recent studies^53^. While the γ-phase is also electroactive, it typically exists in smaller fractions compared to the β-phase^54^. The electroactive β-phase and γ-phase have vital effect on the piezoelectric properties of PVDF, as the β-phase is crucial for optimal performance in piezoelectric applications^55,56^. The orientation and volume of the β-phase significantly impact the piezoelectric and pyroelectric capabilities of PVDF.

The fraction of the electroactive phase was calculated and shown in Table 1, using the following equation derived from the Beer–Lambert law:

5 $$F\left(\beta \gamma \right)=\frac{{A}_{\beta }\gamma }{1.26{ A}_{\alpha }+ {A}_{\beta \gamma }}$$

where F(βγ) is the electroactive phase content fraction, ${A}_{\alpha }$ and ${A}_{\beta\upgamma }$ correspond to absorbance intensities of 760 cm^−1^ and 840 cm^−1^, respectively.

As shown in Table 2, the fraction of electroactive phase content was calculated for samples with different concentrations of GO. The pure PVDF nanofibrous membrane achieved a percentage of 57.49% while this percentage increased when the GO was added to the polymer solutions to reach 68.08% for 1.5 wt.% of GO. The resultant fraction of the electroactive β-phase and γ-phase content shows that 1.5 wt. % is the optimum concentration for the addition of the GO to the PVDF polymer solution to fabricate the desired nanofiber membrane with the highest electroactive β-phase and γ-phase content.

**Table 2:** The electroactive β-phase and γ-phase content of PVDF-GO samples with different GO concentrations.

GO concentration (wt.%)	F(βγ) content %
0	57.50
0.5	47.29
1.0	48.70
1.5	68.13
2.0	40.15
2.5	35.06
3.0	43.85

It is noteworthy that the variations in PVDF-GO nanofiber diameters are correlated with this trend in electroactive phase content. The nanofiber diameter first reduces when the GO concentration rises from 0 wt.% to 1.5wt.%, reaching a minimum at that point. The decrease in nanofiber diameter may probably be linked to GO impact on solution conductivity, which leads to increased fiber pull in the electrospinning procedure. However, the nanofiber diameter starts to rise over the 1.5wt.% GO optimum concentration. This might be explained by the saturation effect, in which adding more GO could cause aggregation or uneven dispersion within the polymer matrix, which would interfere with the electrospinning process and produce thicker nanofibers. The electroactive content fluctuations are probably the result of the intricate interaction between the electrospinning parameters, solution conductivity, and GO concentration^57,58^. Some PVDF-GO mats show a modest reduction in the electroactive content, and this is similar to the result recorded in literature^59^.The whole rotation of the PVDF chain to create the β-phase may be inhibited by the GO sheet itself or by the hydrogen link between the hydroxyl group of GO and PVDF hence, it might be the cause of the β-phase reduction^60^.

### Mechanical characterization

**Figure 9:**
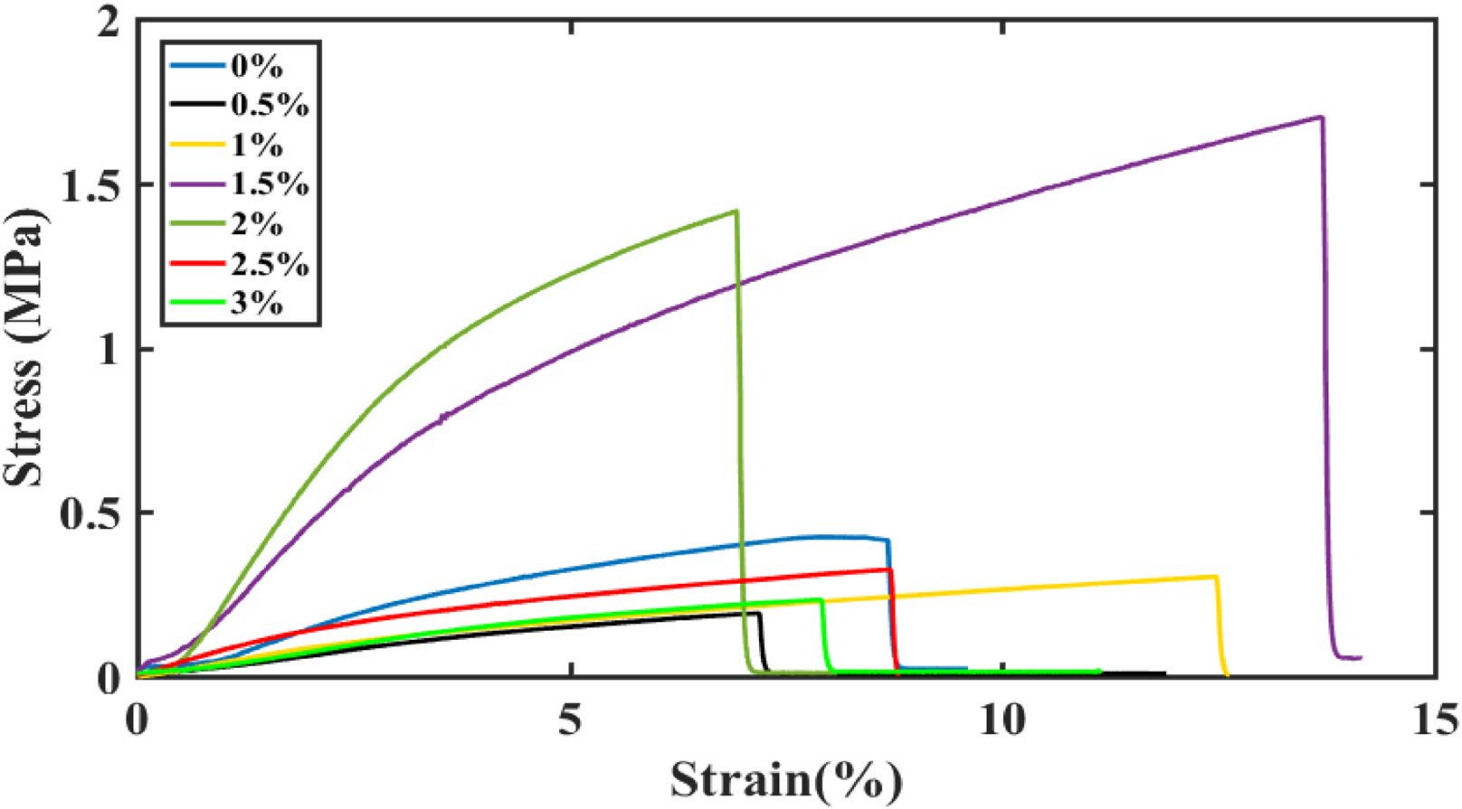
Stress-strain curves for PVDF-GO samples with different GO concentrations.

The mechanical properties of the generated membrane were characterized using tensile stress testing, and the resulting stress-strain curve is presented in Fig. 9. The fabricated samples provide gradual increase in maximum elongation about 8.67%,12.48% and 13.67% for pure PVDF, PVDF-GO 1wt.% and PVDF-GO 1.5wt.%, respectively with tensile stress of 0.41MPa, 0.3MPa, and 1.7MPa, respectively. This significant enhancement in tensile properties is related to several factors. GO nanosheets have very sophisticated mechanical properties^61,62^. In addition, the compatibility between PVDF and GO which is presumably caused by oxygen containing groups which hinders the mobility of polymers chains^63^. In contrast, unexpected behavior is noticed at higher concentration of GO as maximum elongation comes down again to 8.69% and 7.91% for PVDF-GO 2.5wt.% and PVDF-GO wt.3% with tensile stress 0.33 MPa and 0.23 MPa, respectively. And this reversed behavior can be possibly associated to dispersion problems and loss of cohesion between the polymer and nanosheets^64–66^. Overall, the results shown in Fig. 9 demonstrate that adding GO nanosheets with optimum concentration around 1.5 wt.% enhances PVDF mechanical behavior with good tensile strength and young’s modulus compared to piezoelectric nanogenerators^67,68^ before making it more brittle at higher concentrations.

### Piezoelectric analysis

#### Impact test analysis

In this section, we are going to discuss the results of several types of investigation like impulse, frequency, charging, d_33_ test, and sawyer tower analysis to examine piezoelectric response for PVDF-GO nanofibers with different concentrations which gained after we introduce sonication to GO that known to break down GO into few layers^69^. This process achieves good cohesion between PVDF-GO and this manage us to fabricate nanofiber with higher concentrations up to 3 wt.% and that could not be achieved as reported by Hongyan^29^.

**Figure 10:**
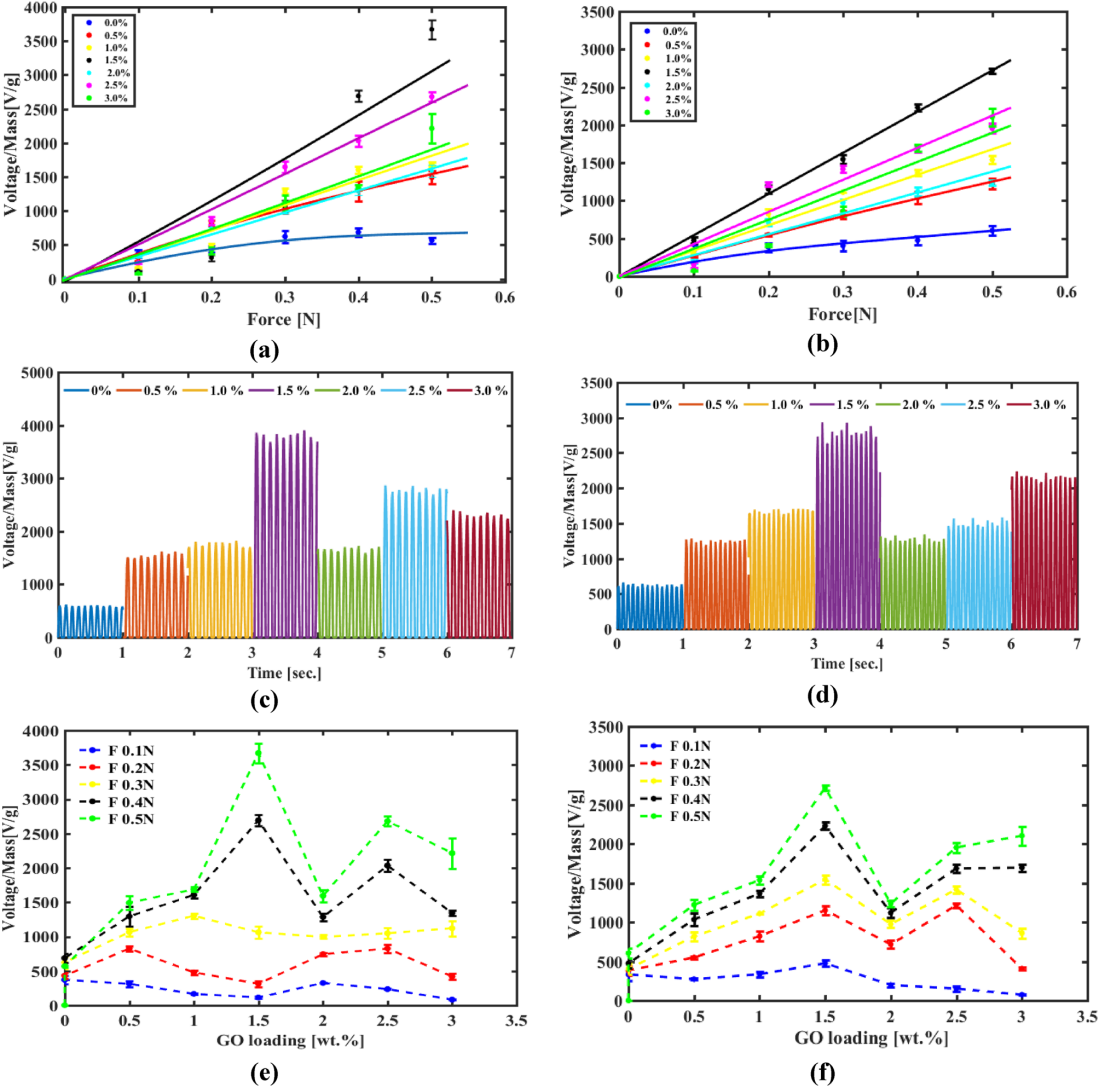
The impulse response for PVDF-GO (**a**,**c**,**e**), and (**b**,**d**,**f**) at (10 Hz, and15Hz) respectively.

Impulse test is performed by applying periodic forces with two different frequencies (specifically 10, and 15 Hz). The shown results in Fig. 10-a,c indicate the enhancement of pure PVDF for diffenet concentrations of GO. The pure PVDF open circuit voltage is (567 V/g) for 0.5 ± 0.01 N at 10 Hz and it is upgraded gradually to 1492 V/g and 1687 V/g for 0.5 wt.% and 1 wt.%, respectively. While, it records 3671 V/g as highest one for GO 1.5 wt.% and also 2716 V/g for 15 Hz too as obvious in Fig. 10-b,d. On the other side, a fair agreements with previous results, piezo response recorded low output for concentrations higher than optimum where open circuit voltage starts to go down again to 2680 V/g and 2213 V/g for 2.5wt.% and 3wt.% repectively. Also, Fig. 10-e,f shows the higher output peaks for GO 1.5 wt. % and the curve is approximately looks like bill shape as piezo response is enhanced until its optimum concentrations then becomes worse compared to pure PVDF.

**Figure 11:**
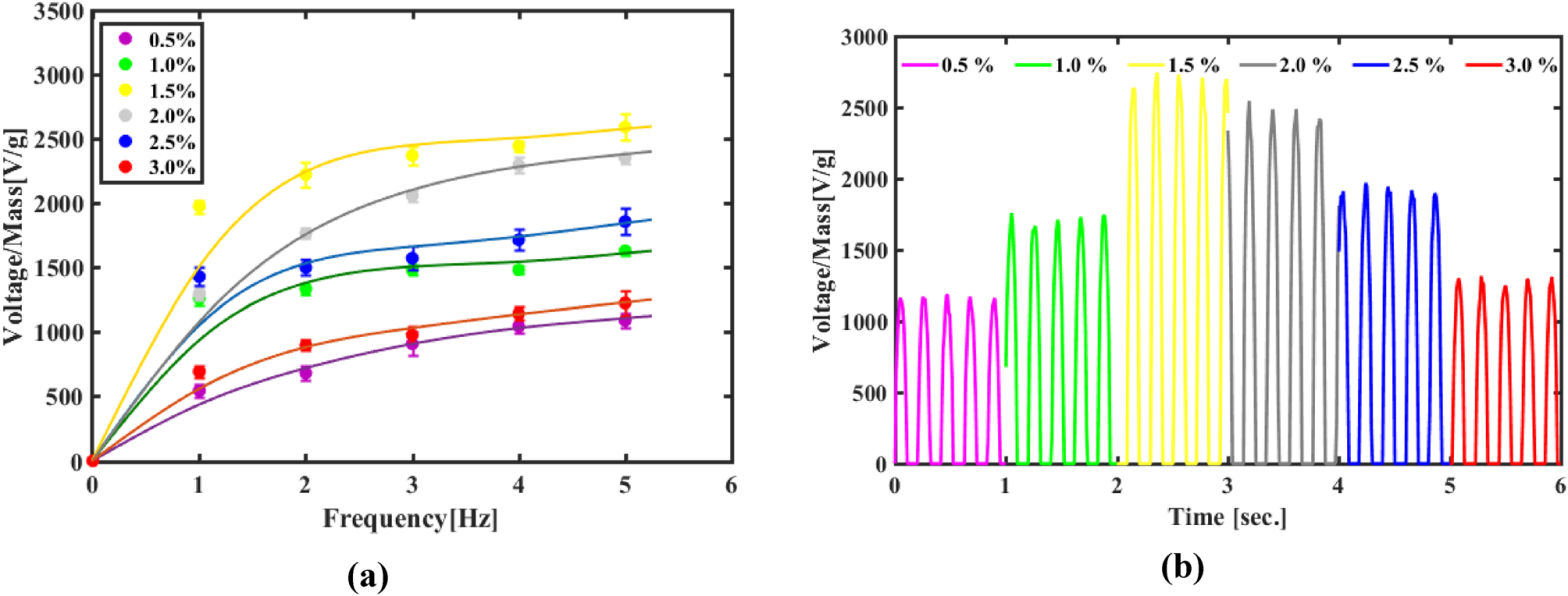
(**a**)The frequency response for PVDF/GO wt.% at force 0.3 N, (**b**) wave pulses output at 5Hz and 0.3N.

Also, this behavior is ensured in the frequency test as we change frequency from 1 to 5 Hz while force is constant at 0.3 N. As presented in Fig. 11, the output goes up linear before it saturates approximately after 2 Hz . Again, an increase in output voltage as GO wt. % increase is noticed. It is measured as 1091 V/g and 1630 V/g for 0.5 wt.% and 1 wt.% then it decreases again with GO increment after going beyond optimun concentration which is 1.5 wt.% that generates 2593 V/g then it goes back to 1857 V/g and 1224 V/g for 2.5 wt.% and 3 wt.%. This finding is in line with our previous results regarding the optimum concentration 1.5 wt.%.

#### Analysis of piezoelectric coefficient, d_33_

**Figure 12:**
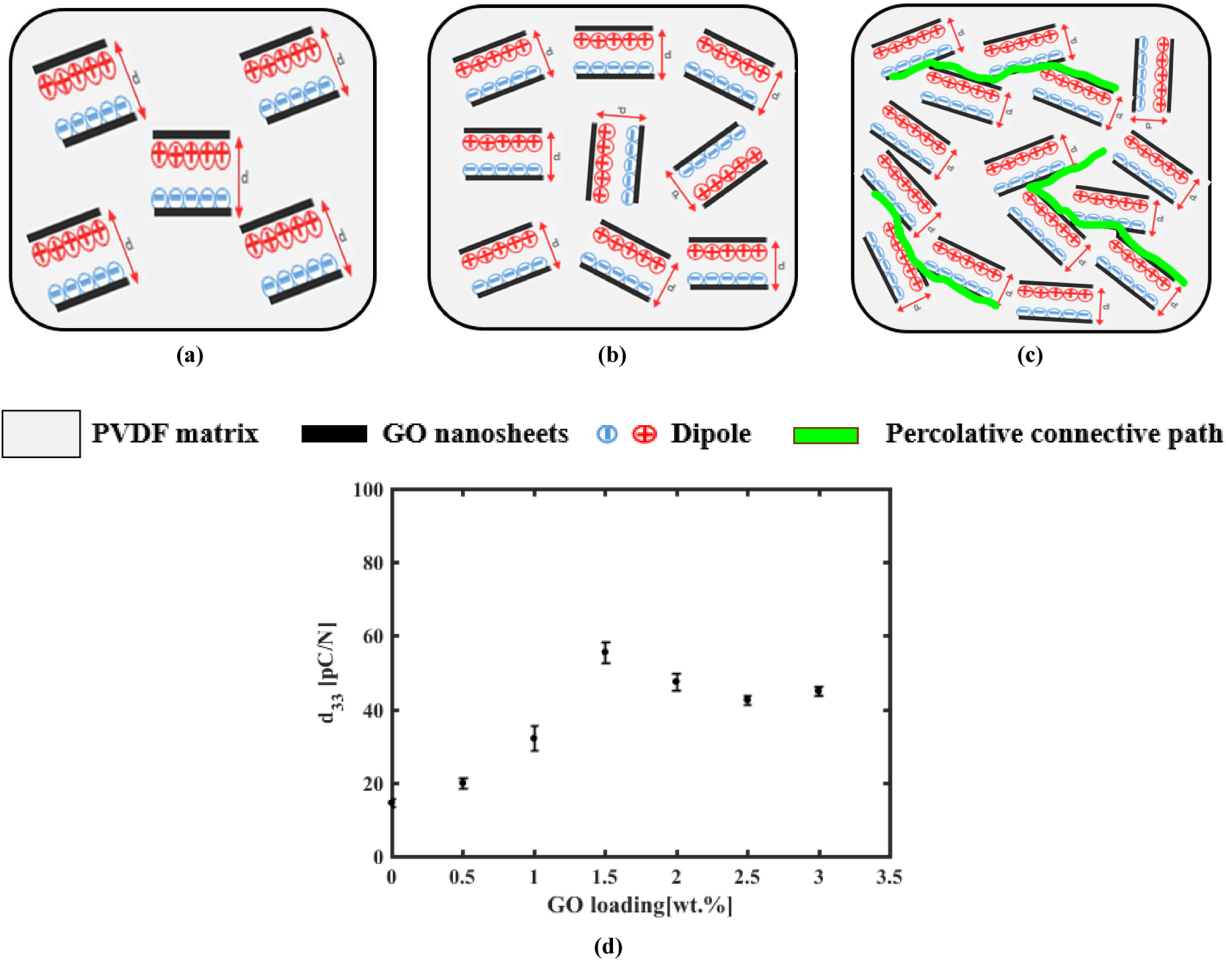
Schematic of micro-capacitors concentration inside PVDF-GO wt.% composite nanofiber with different concentration (**a**) below GO 1.5 wt.%, (**b**) for GO 1.5wt.%, (**c**) above GO 1.5wt.%, and (**d**) d_33_ results for PVDF-GO wt.%.

The piezoelectric coefficient d_33_ is calculated according to Eq. (6)^28^

6 $${d}_{33}=\frac{Q}{F}$$

where F is the applied force and Q is the charge. To have a good explanation about d_33_ results we applied constant force (~ 250 mN) deliberately using Sinoceramics piezo d_33_ test system to ensure that d_33_ is proportional with the charge. GO addition process forms micro-capacitors as shown in Fig. 12-a where GO acts like parallel conductive plates and PVDF as the insulator^60^. Looking for d_33_ test outcome in Fig. 12-d, that reveals d_33_ coefficient enhancement with GO addition to PVDF with respect to pure PVDF that recorded 14.57 pC/N. Moreover,it records 19.94 pC/N and 32.07 pC/N for 0.5 wt.% and 1 wt.%, respectively. This enhancement is attributed to accumulation of charges in micro-capacitors.As the concentration of GO increases,the formation of micro-capacitors increases^70^. And so, it increases d_33_ too as indicated in Fig. 12-b. It is obvious that the best d_33_ coefficient is 55.57 pC/N for PVDF-GO 1.5wt. % and it goes back for higher concetrations as observed where d_33_ coefficient is 47.53 pC/N and 42.53 pC/N for 2 wt.% and 2.5 wt.%, respectively. This diminution of d_33_ is attributed to decreasing in accumulated charges where GO higher concentration forms conducting paths across the sample as shown in Fig. 12-c. Because of percolative network paths of GO nanosheets due to higher concentrations, charges flow across the sample rather than accumulation inside it^71^. The average values of d_33_ are shown in Table 3. It is notethably, d_33_ coefficient goes back after ideal concentration as mensioned before and these results have fair matching with previous impact and frequency response, which is the more alignment of dipoles according to optimum added concentration of GO. It is obvious that sonication treatment has good impact to enhance d_33_ coefficient for the same concentration approximately compared to result has been reported in literature^28^**.**

**Table 3:** Average d_33_ of PVDF-GO samples with different concentrations of GO.

GO concentration (wt.%)	d_33_ [pC/N]
0	14.54 ± 0.94
0.5	19.94 ± 1.43
1.0	32.07 ± 3.41
1.5	55.57 ± 2.88
2.0	47.53 ± 2.18
2.5	42.53 ± 1.14
3.0	45.02 ± 1.32

#### Charging capacitance

**Figure 13:**
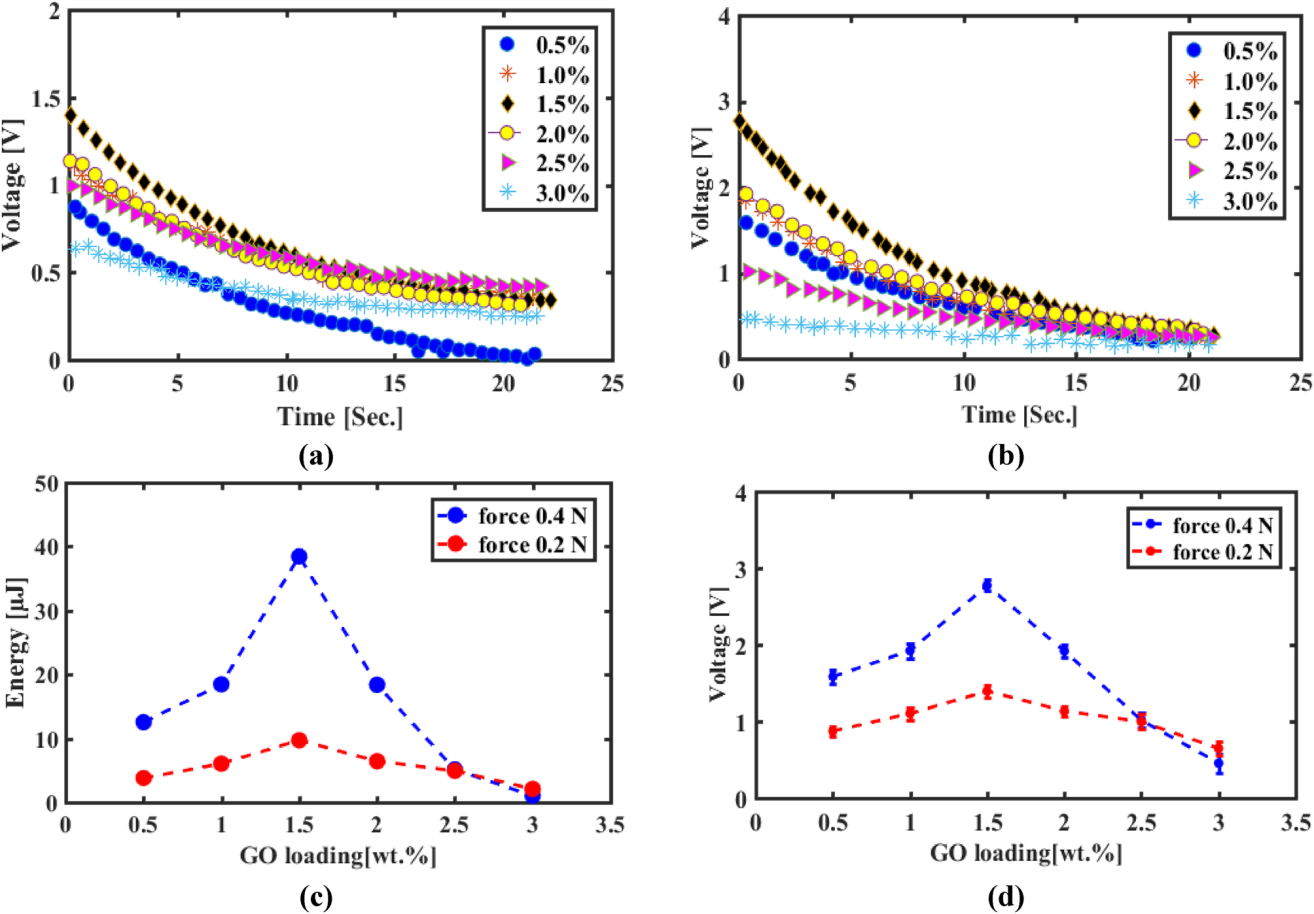
Quantitative characterization of charging and discharging for PVDF-GO wt.%. (**a**) discharging curves due to 0.4N impact force, (**b**) discharging curves due to 0.2N, (**c**) variation of maximum charging voltage at 0.2N and 0.4N, and (**d**) variation of charged energy at 0.2N and 0.4N.

Charging capacitance test is performed as a vital step to ensure the performance of the nanogenerator and the capability of energy harvesting process. The test is performed twice at two loads 0.2 N and 0.4 N where the nanogenerator is linked to test circuit and results are indicated in Fig. 13. The discharging process of charge on capacitor (due to generated voltage) from the maximum level indicates the value of harvested energy that released through resistive load. Hence, looking at the results, we find that they confirm and are consistent with what was found in previous tests, as the concentration of GO 1.5 wt. % is considered the best one, recording maximum open circuit voltage of 1.395 V and 2.773 V for both loads, respectively. By calculating the energy stored during the charging process according to Eq. (2), we find that maximum energy in the range 9.729 µJ and 38.46 µJ.

**Figure 14:**
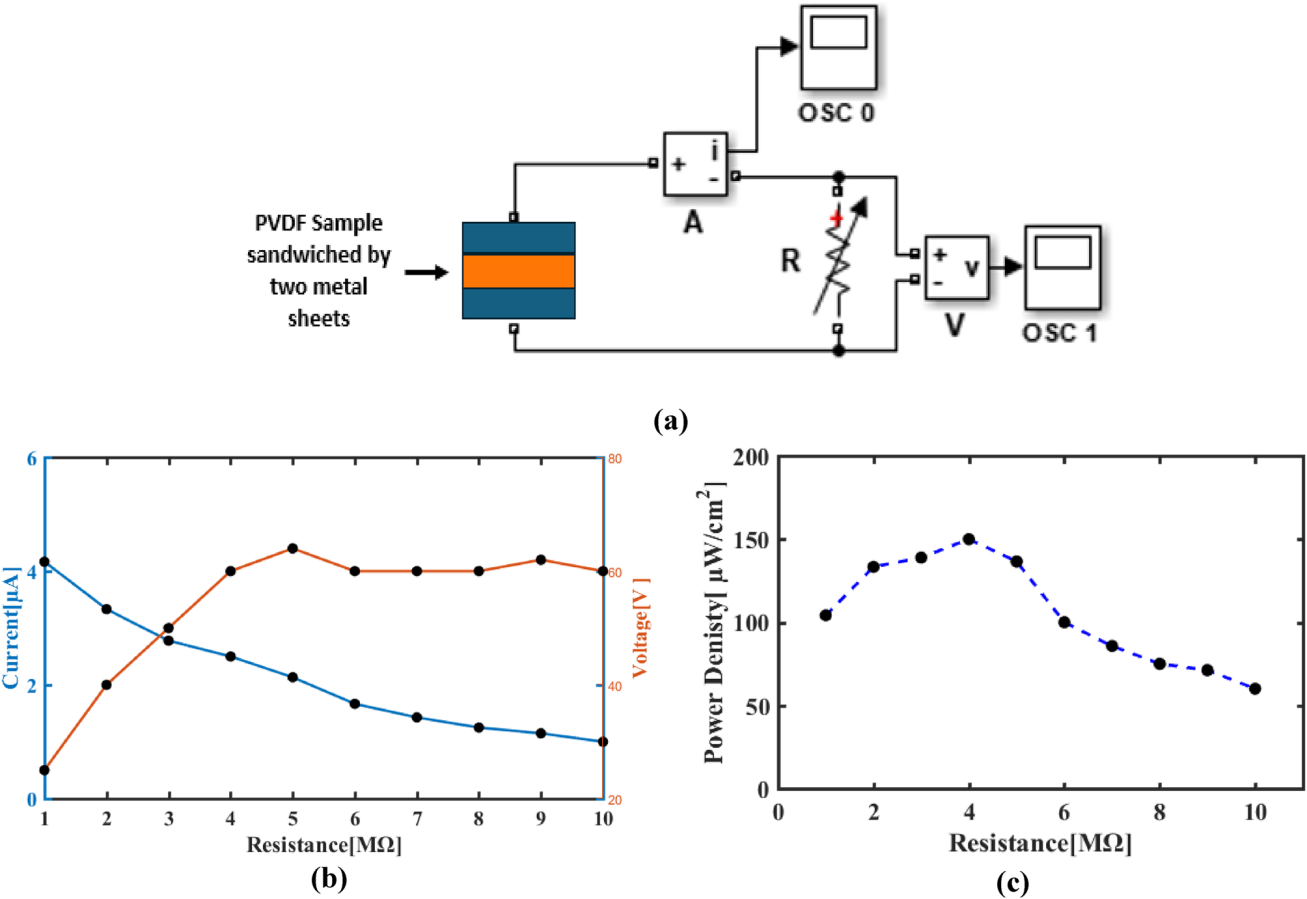
(**a**) Schematic circuit used to evaluate PVDF-GO 1.5 wt.% Ohmic behavior, (**b**) Voltage and current output as a function of load resistance, and (**c**) Power density as a function of load resistance.

Figure 14-b displays the voltage and current output signals across various load resistances (R_L_) for a PVDF-GO 1.5 wt.%. sample. The findings were acquired via measuring the output voltage by applying pressure in the impact mode at (0.4 N—10Hz) across a range of load resistances, from 0.1 to 10 MΩ. The schematic in Fig. 14-a displays the relevant circuit that was utilized. With an increase in load resistance, the output voltage of the nanogenerator rose sharply but its current output progressively declined (Fig. 14-b). It is noteworthy to clarify that 2.773 V indicated in Fig. 13-b is the maximum open circuit voltage recorded on capacitor due to accumulation of charges produced during charging process by 0.4 N impact load. On the other hand, 60V revealed in Fig. 14-b is almost instantaneous open circuit pulses voltage due to impact load performed in power density test at high resistance ~ 10 MΩ. At resistance to higher values, there is a little drop in output voltage was noted. Equation (7) was used to calculate the instantaneous power density (P_d_) of the PVDF-GO 1.5wt.% nanogenerator:

7 $${P}_{d}=\frac{{V}^{2}}{A {R}_{L}}$$

where V is the output voltage, and A is the PVDF-GO nanogenerator effective area. The maximum power density indicated by the PVDF-GO nanogenerator was 150µw/cm^2^ at 4 MΩ as illustrated in Fig. 14-c.

#### Analysis of sawyer—tower circuit

**Figure 15:**
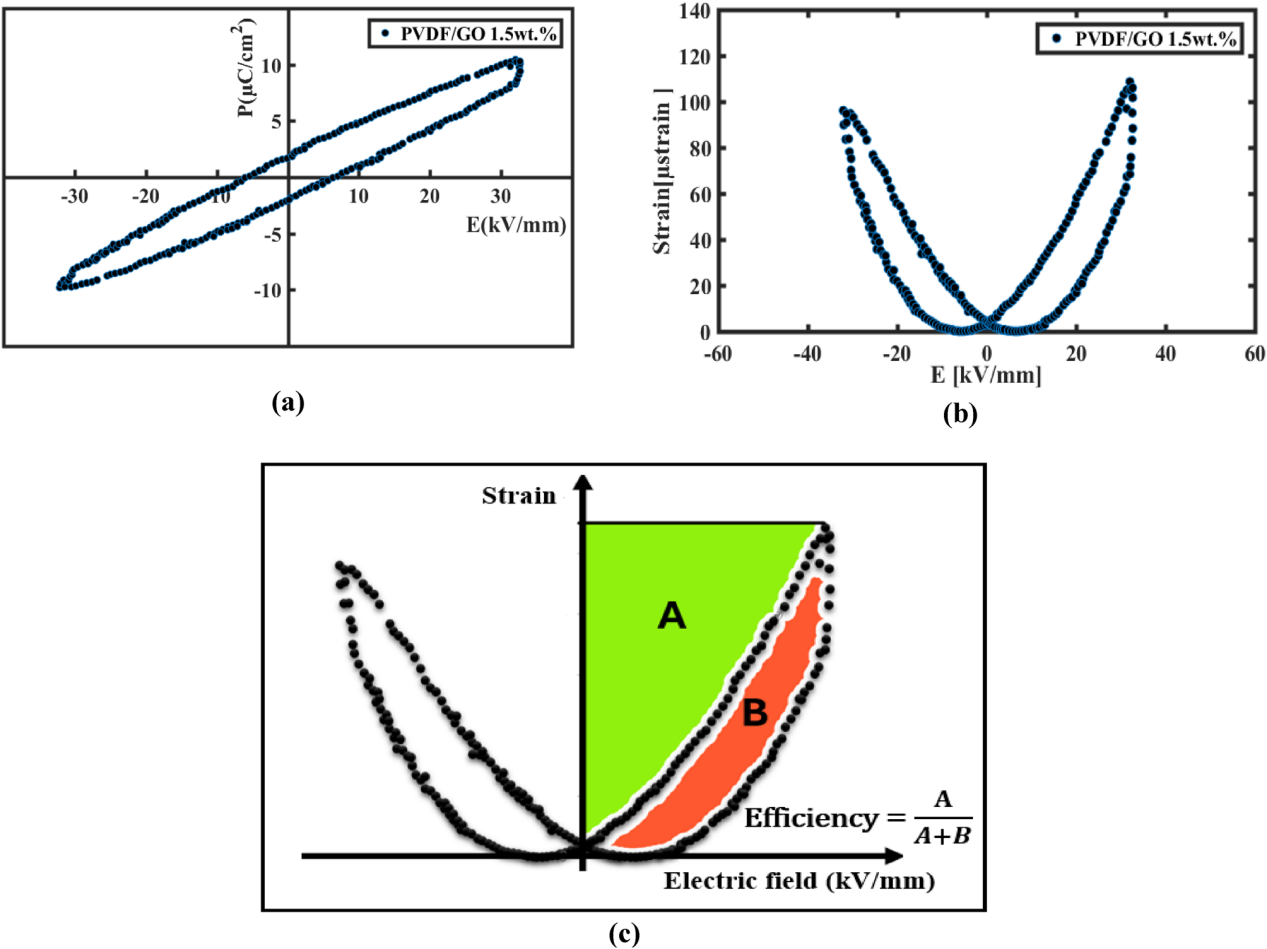
(**a**) P–E Hysteresis loops for PVDF-GO 1.5wt.% (**b**) S–E hysteresis loops for PVDF-GO 1.5wt.% (**c**) Electro-mechanical efficiency criteria.

The Sawyer–Tower circuit is a simple electronic circuit used to measure the electrical hysteresis loop and polarization of ferroelectric materials providing a wealth of information about the material being tested such as linear permittivity that could be measured from P–E curve which equal the slope of saturation region^72^. A sinusoidal waveform voltage is applied to the circuit, and the resulting P–E curves are indicated. Then, S–E curves are obtained to ensure our previous results and determine the energy storage efficiency as well as the electromechanical conversion efficiency of PVDF-Go nanogenerator. The area enclosed by curve represents losses even in P–E and S–E curves so we get data about electromechanical conversion efficiency that calculated through the criteria proposed by Faizan and as presented in Fig. 15-c^73^. Figure 15-a reveals PVDF-GO 1.5 wt.% properties such as remanent polarization (Pr) and coercive field (Ec). Furthermore, energy storage efficiency is up to 72.16%. As well as Fig. 15-b obviously clarifies electromechanical properties of PVDF-GO 1.5 wt.% via S–E curves. Moreover, electromechanical conversion efficiency is about 74.73%. Table 4 summarized all data about PVDF-GO 1.5 wt.% and shows that efficiency for GO loading is moderate compared to piezoelectric energy harvesters^74^. In addition, Table 4 reveals a well-alignment with all previous piezo-response results.

**Table 4:** Ferroelectric parameters of PVDF/GO 1.5 wt.% composite nanofiber.

Remanent polarization (Pr)	Coercive field (Ec)	Energy storage efficiency %	Electro-mechanical efficiency %
2.136 µC/cm^2	6.329 kV/mm	72.16	74.73

## Conclusion

In this extensive study, we investigated the piezoelectric and mechanical properties of electrospun PVDF nanofibers with different GO concentrations up to 3 wt.% through comprehensive numerous tests to enhance energy harvesting performance. We had deliberately long-time interval about five months for PVDF/GO samples fabrication and testing process with suitable time intervals between each test to ensure that the output is stable, and all results are in line. We noted the vital role of graphene oxide (GO) as a nucleation agent to boost the electroactive β-phase and γ-phase content. We mainly examined the impact of GO different concentration on microstructural, mechanical, and piezoelectric properties. The study of GO impact on PVDF reveals that GO should be added in an optimum quantity for any fabrication method due to micro-capacitors formation phenomenon. GO addition has bill shape enhancing effect around optimum concentration. In our study while adding GO, the PENG performance is enhanced until reaching the optimal concentration of 1.5 wt.%, beyond which a significant decline is noted. With higher GO content, fiber’s average diameter became larger with worse uniformity. Among the concentrations, PVDF-GO 1.5 wt.% achieved electroactive phase content ~ 68.13% and generated open circuit voltage ~ 3671 V/g. A maximum output power density ~ 150 µw/cm^2^ was achieved at 4 MΩ and a conversion efficiency is ~ 74.73%. Furthermore, the improved mechanical properties showed the ability to be used in wearable electronics. This research advances Piezoelectric nanogenerator (PENG) technology, emphasizing the potential of PVDF for future development.

## Data Availability

The datasets used and/or analyzed during the current study are available from the corresponding author on reasonable request.
